# Recent advances in understanding/managing trigeminal neuralgia

**DOI:** 10.12688/f1000research.16092.1

**Published:** 2019-04-17

**Authors:** Mark Obermann

**Affiliations:** 1 Center for Neurology, Asklepios Hospitals Schildautal, 38723 Seesen, Germany; 2 Department of Neurology, University of Duisburg-Essen, Hufelandstr. 55, 45122 Essen, Germany

**Keywords:** trigeminal neuralgia, facial pain, recent advances, pathophysiology, treatment options, future treatment

## Abstract

Despite recent advances in understanding and treating trigeminal neuralgia, its management remains a considerable challenge. Better classification of different types of facial pain and the identification of prognostic factors for different treatment options lead the way toward better quality of life for the individual patient. Although the principles of treating trigeminal neuralgia remain basically the same, antiepileptic drugs, muscle relaxants, and neuroleptic agents are widely used medical treatment options. They were not originally developed for treating trigeminal neuralgia. Carbamazepine was studied in adequate placebo-controlled clinical trials in the 1960s and is still considered the most effective drug. Among emerging treatment options currently under clinical investigation are local botulinum neurotoxin type A injections and a novel sodium channel blocker (CNV1014802) that selectively blocks the Na _v_1.7 sodium channel. Non-pharmacological treatment options are non-invasive electrical stimulation with either transcranial direct-current stimulation or repetitive transcranial magnetic stimulation which both require further evaluation in regard to applicability. Surgical options remain a valid choice for patients not responding to medical treatment and include Gasserian ganglion percutaneous techniques, gamma knife surgery, and microvascular decompression. There is continual effort to improve these techniques and predict the outcome for better patient selection.

## Introduction

Trigeminal neuralgia (TN) is defined by the International Headache Society as a “unilateral disorder characterized by brief electric shock-like pains, abrupt in onset and termination, and limited to the distribution of one or more divisions of the trigeminal nerve” ^1^. The revised ICHD-III (International Classification of Headache Disorders, 3rd edition, beta version) classifies TN as classic (essential or idiopathic) TN without or with concomitant persistent facial pain ^1, 2^. TN caused by trauma, tumor, herpes zoster, or multiple sclerosis is classified as secondary painful trigeminal neuropathy. A slight hyperesthesia or hypoesthesia, either of which is often present, is now in line with the classic TN diagnosis ^2^.

TN typically starts in the second or third branches of the trigeminal nerve ^2^. An involvement of the ophthalmic nerve may be associated with other differential diagnoses such as severe unilateral neuralgiform headache attacks with conjunctival injection and tearing (SUNCT) and luckily is present in less than 5% of cases ^3^. Typical TN attacks usually last between 1 second and a few seconds. TN may also occur in clusters of different duration and intensity lasting up to 2 minutes. The attack is followed by a short refractory period in many patients, during which a new attack cannot be evoked by further stimulation ^4^. In between paroxysms, patients are usually pain-free; however, in some patients, a dull, concomitant background pain may persist ^2^. Pathophysiological mechanisms explaining this concomitant pain are missing, but it seems to be associated with a poor medical and surgical outcome ^5– 8^. Although many patients respond to first-line therapy initially, most treatment approaches tend to lose efficacy over time, so new and innovative treatment options are warranted. Many patients will receive surgery after their medical treatment failed, whereas others require medical treatment after the long-term efficacy of surgery slowly deteriorates.

## Quality of life

Severe attacks may cause an inability to speak or eat. Many patients contemplate the constant fear of pain that may return at any time even between attacks ^9^, which results in serious impairment of their individual daily function and their quality of life. Reduced measures of daily functioning, quality of life, well-being, sleep, mood, and overall health status correlated with pain severity ^10^. In 34% of patients with TN, even employment was impacted. Depression is quite frequent in this patient population. Moderate to severe pain was reported by up to two thirds of patients within the previous 24 hours ^10^. Owing to difficulties in properly assessing the burden of disease, a recent study evaluated the validity of a revised version of the Penn Facial Pain Scale (Penn-FPS-R), which focuses on the patients’ health-related quality of life. The Penn-FPS was introduced as a supplement to the Brief Pain Inventory Pain Interference Index (BPI-PII) and was revised to include a total of 12 items with more TN-specific additions such as “biting and chewing”, “self-care” (brushing the teeth, shaving, and so on), and “temperature change” (moving outside, air-condition effects, and so on). Besides the usefulness of the questionnaire, this study revealed a high burden of disease with an average mean pain of 6.85 (standard deviation of 2.34) on a 0- to 10-point numerical rating scale despite adequate treatment attempts in the majority of included patients ^11^.

## Prognostic factors

A correct diagnosis is the paramount factor for adequate treatment and thus a good outcome. Differentiating trigeminal autonomic cephalalgias (for example, cluster headache, SUNCT, and paroxysmal hemicrania) and other craniofacial pain syndromes or persistent idiopathic facial pain is very important, as treatment is fundamentally different. Patients with first-division trigeminal pain only may have symptomatic TN (STN) (that is, due to multiple sclerosis or tumor) ^12^, which is more difficult to treat ^13, 14^. Routine head imaging with magnetic resonance imaging (MRI) can detect structural causes in as many as 15% (95% confidence interval [CI] 11–20%) of patients excluding those with microvascular conflict. The blink reflex and other trigeminal reflex tests have a high accuracy to identify patients with STN. Five independent studies showed a pooled sensitivity of 94% (95% CI 91–97%) and a pooled specificity of 87% (95% CI 77–93%). Evoked potentials were unable to sufficiently distinguish classic TN from STN (pooled specificity 64%, 95% CI 56–71%; pooled sensitivity 84%, 95% CI 73–92%) ^15, 16^.

MRI plays a major role in the pre-surgical assessment in order to determine the presence of microvascular conflict. Specificities and sensitivities are variable (specificity 29 to 93%, sensitivity 52 to 100%) between studies and this is probably related to different MRI sequences used in different investigations ^15, 16^. Moreover, a large imaging study of 135 patients with TN showed the presence of neurovascular conflict (NVC) on the symptomatic and the asymptomatic side (89% versus 78%, *P* = 0.014; odds ratio 2.4, 95% CI 1.2–4.8, *P* = 0.017). Severe NVC was more frequent on the symptomatic side compared with the asymptomatic one (53% versus 13%, *P* <0.001; odds ratio 11.6, 95% CI 4.7–28.9, *P* <0.001). The most common causes for severe neurovascular contact were arteries in 98% of cases ^17^. NVC causing atrophy or displacement of the nerve (or both) was highly associated with pain on the symptomatic side of patients with classic TN in contrast to NVC in general, according to this study ^17^. A more recent study reported that neurovascular contact with morphological changes to the nerve as detected by 3-Tesla MRI and male gender was associated with better outcome following microvascular decompression surgery ^18^. Excellent outcome and concomitant persistent pain, current age, or disease duration were not correlated ^18^. Outcome after microvascular decompression surgery was only slightly worse; recurrence rates were 9.23% in the age group younger than 65 years and 13.33% in the group older than 65 years ^19^. However, long-term outcome was determined by concomitant pain as these patients developed TN recurrence in 60.3% following microvascular decompression surgery whereas patients without concomitant pain did not show signs of recurrence in 91.8% within a mean follow-up period of 20.6 months ^20^. Depression and anxiety, along with a deterioration in quality of life, are common in patients with TN ^21^. About 2% of patients with multiple sclerosis were reported to have symptoms similar to those of patients with TN ^3^. TN commonly runs in families, but there have been reports of an increased risk in people living in the same household, which suggests that environmental factors may influence the disease ^22, 23^. Genetic variants of TN were also suggested in two investigated families: autosomal dominant in one family and autosomal recessive in the other ^24^.

## Developing treatment options

A novel substance finished its phase II trial last year with promising results. BIB074 (formerly known as CNV1014802, proposed name vixotrigine, formerly raxatrigine) is a new state-dependent sodium channel blocker with potency and selectivity of the Na _v_1.7 sodium channel over the different tested subtypes (Na _v_1.1, Na _v_1.2, Na _v_1.3, Na _v_1.5, Na _v_1.6, and TTX-R) for the depolarized and resting states. Sodium channel blocking quantity increases in parallel to increased stimulation frequency of Na _v_1.7 and of Na _v_1.2 and Na _v_1.6. BIB074 preferentially targets and inhibits higher frequencies (10 Hz and more) induced during seizures or by noxious stimuli ^25^.

A novel randomized withdrawal design was used in the phase II study in order to show its efficacy ^26^. A 21-day open-label treatment period using BIB074 150 mg three times per day (tid) was followed by randomization into a double-blind treatment phase of 28 days with either placebo or BIB074 150 mg tid only in those patients who showed a successful treatment response within the final week ^27^. The others were considered non-responders and dropped out of the study in order to go back on their previous TN medication. Thirty percent or more reduction in pain severity as well as a 30% reduction in numbers of paroxysms relative to the run-in period was defined as treatment response. Sixty-seven patients were included in the study and 69% completed the open-label phase to enter the double-blind phase.

BIB074 was able to reduce the number of paroxysms and the overall pain severity in all primary and secondary outcomes but missed statistical significance in its primary endpoint. A reduction of the number of paroxysms by 60% compared with 12% for placebo was demonstrated, and pain severity decreased by 55% compared with 18% under placebo treatment. The primary endpoint was treatment failure rate of 33% with BIB074 compared with 65% with placebo and a satisfactory separation of both conditions on the Kaplan–Meier time to relapse. BIB074 was well tolerated and no serious adverse events related to the drug were reported. The adverse event profile was comparable to that of placebo in the double-blind phase of the study ^27^. The results are quite promising, but it should be remembered that the patient numbers were low and there was a short evaluation period, so this novel therapeutic option must prove its efficacy over time. A multicenter and international phase III study by Biogen is planned.

Botulinum neurotoxin type A (BoNT-A) was effective in the treatment of TN in recent studies. The pharmacological mechanism remains unresolved but includes the local release of anti-nociceptive neuropeptides such as glutamate, substance P, and calcitonin gene-related peptide (CGRP) in order to reduce central and peripheral sensitization ^28^. Significant symptom relief following BoNT-A injections was shown in a small uncontrolled clinical trial (N = 13). BoNT-A was administered at a mean dose of 3.22 units/cm ^2^ subcutaneously directly in the painful area of the face. The treatment effect of BoNT-A slowly faded after 60 days ^29^. A randomized, placebo-controlled, double-blind study investigated 84 patients with classic TN treated with 25 U or 75 U of BoNT-A: placebo (n = 28), BoNT-A 25 U (n = 27), BoNT-A 75 U (n = 29). The duration of the study was 8 weeks. Pain severity, efficacy, and side effects were the endpoints of the study. Significant reduction of pain was shown on a visual analogue scale with both the 25 U and 75 U groups compared with placebo after 1 week, which remained stable for the whole study period. Responders among the 25 U group (70.4%) and 75 U group (86.2%) were more common compared with placebo (32.1%) at week 8. No difference between the 25 U and 75 U groups was detectable. Patient Global Impression of Change (PGIC) showed that 66.7% (25 U group) and 75.9% (75 U group) of patients reported “much improved” or “very much improved” pain symptoms but that only 32.1% of the placebo patients stated this outcome. Only mild or moderate side effects were documented ^30^. The authors of a recent comprehensive meta-analysis showed a pooled reduction of −3.009 points on a 0 to 10 verbal rating scale (95% CI −4.566 to −1.453, *P* <0.001) after treatment with BoNT-A and confirmed moderate evidence for the efficacy of BoNT-A ^31^. These promising findings will have to be confirmed by additional controlled clinical trials in order to recommend the use of BoNT-A for the treatment of TN ^32^.

## Established medical treatment

Pharmacological and surgical treatment options are numerous, widely used, and not seldom efficacious ( Table 1). Medical therapy should be the first choice, and only after two failed treatment attempts may surgical interventions be considered in patients. Between 33 and 50% of patients may require surgical intervention at some point. No direct comparison studies between medical and surgical treatment exist. Active support group participation may help patients to better cope with their condition and stay compliant with medication ^33^.

**Table 1.** Therapeutic options in trigeminal neuralgia.

First line	Carbamazepine (600–1200 mg/day) or oxcarbazepine (600–1800 mg/day)
Second line	Add-on or switch to lamotrigine (400 mg/day) Baclofen (40–80 mg/day) Pimozide (4–12 mg/day)
Surgery	Percutaneous procedures on the Gasserian ganglion Percutaneous glycerol rhizolysis Radiofrequency thermocoagulation Balloon compression Gamma knife radiosurgery Microvascular decompression
Alternative medical treatment options (class III or IV)	Pregabalin (150–600 mg/day) Gabapentin (900–3600 mg/day) Topiramate (100–400 mg/day) Tocainide (20 mg/day) Valproate (600–2400 mg/day)

Carbamazepine (CBZ) (200–1200 mg/day) and oxcarbazepine (OXC) (600–1800 mg/day) should be considered first-line therapy, according to commonly accepted treatment guidelines ^15, 16^. Even though CBZ has stronger evidence ^34– 37^, OXC is generally considered to be better tolerated ^38^. Second-line therapy includes add-on therapy with lamotrigine (400 mg/day) ^39^, change to lamotrigine monotherapy, or the use of baclofen (40–80 mg/day) ^40^. Pimozide (4–12 mg/day) is seldom in clinical use. Different antiepileptic drugs were investigated in open-label studies with small patient numbers. Efficacy was described for clonazepam, pregabalin, gabapentin, phenytoin, topiramate, valproate, and tocainide (12 mg/day) ^41^.

## Neuromodulation techniques

Neuromodulation offers an alternative worth considering for patients whose neuropathy pain is refractory to pharmacotherapy. Central and peripheral neuromodulation are available, but the clinical evidence base is very limited. Options include electrical Gasserian (trigeminal) ganglion stimulation ^42^, peripheral nerve stimulation ^43, 44^, and invasive motor cortex stimulation ^45^ and non-invasive cortex stimulation ^46, 47^. Patient self-conducted motor cortex transcranial direct-current stimulation (tDCS) showed excellent efficacy on pain reduction in patients with classic TN. Ten patients received daily stimulation over the course of 20 minutes for a total of 2 weeks with anodal (1 mA) or sham tDCS over the primary motor cortex (M1) in a double-blind, randomized, crossover design. Pain intensity was the primary outcome variable on a verbal rating scale (0–10). Anodal tDCS resulted in a 29% reduction of pain intensity after treatment ( *P* = 0.0008). Reduction in attack frequency was also observed but did not reach statistical significance. No relevant adverse events were reported. Anodal tDCS over the course of two weeks may become a valuable treatment option for patients otherwise unresponsive to standard medical treatment ^8^.

Repetitive transcranial magnetic stimulation (rTMS) is also a relatively novel technology introducing the possibility of testing the responsiveness of patients with trigeminal neuropathic pain to invasive epidural cortical stimulation. In 24 patients, TN was treated with daily 20-Hz motor cortex stimulation over the course of five days. Ratings of pain decreased by 45% for at least 2 weeks ^48^. A different investigation included 12 patients who failed surgery with intractable TN, out of which 58% reported more than 30% reduction of pain intensity following rTMS ^49^.

## Surgical treatment

Medical treatment-refractory patients with a minimum of two adequately dosed recommended TN medications, including CBZ, are candidates for surgical treatment. The patients’ symptoms, not any neuroimaging investigations, are the most relevant factor for this decision ^50^. TN surgical management is either ablative (destructive) with the intentional destruction of sensory function of the trigeminal nerve or non-destructive with mere decompression of the trigeminal nerve and preservation of its normal function.

Percutaneous techniques to the Gasserian ganglion are all destructive and consist mainly of percutaneous glycerol rhizolysis, radiofrequency thermocoagulation, and balloon compression. Pain relief was reported by 90% of patients undergoing these procedures. Approximately 68 to 85% of patients remain pain-free after 1 year but this deteriorates to 54 to 64% after 3 years and only 50% are still pain-free after 5 years. Sensory loss (50%) is the most common side effect with high impact on quality of life for these patients ^21^, followed by dysesthesias (6%), corneal numbness with risk of keratitis (4%), and anesthesia dolorosa (4%). Gasserian ganglion treatments are generally minor, overnight procedures with very low mortality ^15, 16^.

Gamma knife surgery uses a focused radiation beam to sever the trigeminal root in the posterior fossa. Sixty-nine percent of patients were reported to remain pain-free 1 year after gamma knife surgery without additional medication. After 3 years, this was down to 52%. Pain relief may require up to several weeks (mean of 1 month) in order to develop. Sensory complications were reported in 6% of patients with a delay of up to 6 months, including paraesthesias in 6 to 13%, and facial numbness in 9 to 37% that may improve with time ^15, 16^. Quality of life improves by 88% ^21^. However, gamma knife surgery is quite expensive, limiting its more widespread usage. This makes it a treatment reserved for patients unfit to bear conventional surgery or with blood coagulation disease or medication (for example, warfarin).

The most sustained pain relief was reported following microvascular decompression surgery. Ninety percent of patients had initial pain relief. More than 80% were still pain-free 1 year after surgery and this fell to 75% after 3 years and 73% after 5 years. However, it is a major surgical intervention including craniotomy in the posterior fossa to reach the trigeminal nerve. Mortality rates range from 0.2 to 0.5% on average, and about 4% of patients have serious adverse events such as infarcts, hematomas, or cerebrospinal fluid leakage. The most frequent complications are aseptic meningitis (11%), hearing loss (10%), and sensory loss (7%) ^15, 16^.

Recent studies focused on the long-term evaluation of different surgical treatments ^51, 52^ and the improvement of common surgical techniques ^53– 55^. A huge number of studies were conducted in this regard over the past several years, but most of them remain on a descriptive level unable to unveil evidence-based comparisons and therefore inspire only indirect recommendations. It remains to be determined what the right timing for surgical intervention is ^56^. Some experts recommended early surgical referral of patients unresponsive to first-line medical treatments, whereas other experts require at least two different drugs (including CBZ) alone and in combination medical therapy before even considering surgery. Supporting final evidence for either of the two options is still missing. It seems reasonable to refer patients unresponsive to medical treatment for surgical intervention without a long delay.

## Conclusions

Treatment of TN is still challenging, as individual responses to different therapeutic options may vary considerably. Only a few available therapy options have confirmed efficacy fulfilling current standards for evidence-based medicine. However, novel therapeutic options are on the rise; for the first time, substances are in clinical testing on larger patient populations specifically for this very disabling but rare disease. Outcome predictors and risk factors for treatment failure are being systematically assessed more and more so that an individual patient-guided treatment decision can be made. The continual effort by clinicians, researchers, and the pharmaceutical industry may soon provide therapeutic options that are more tolerable, more specific, and more efficient for patients with TN.
